# Seizure outcomes of temporal lobe epilepsy surgery in patients with normal MRI and without specific histopathology

**DOI:** 10.1007/s00701-017-3127-y

**Published:** 2017-03-09

**Authors:** Jugoslav Ivanovic, Pål G. Larsson, Ylva Østby, John Hald, Bård K. Krossnes, Jan G. Fjeld, Are H. Pripp, Kristin Å. Alfstad, Arild Egge, Milo Stanisic

**Affiliations:** 1 Department of Neurosurgery, Oslo University Hospital, Sognsvannsveien 20, N-0027 Oslo, Norway; 2 Clinical Neurophysiologic Laboratory, Department of Neurosurgery, Oslo University Hospital, Oslo, Norway; 3 Department of Clinical Psychology and Neuropsychology, National Centre for Epilepsy, Oslo University Hospital, Oslo, Norway; 4 Department of Radiology, Oslo University Hospital, Oslo, Norway; 5 Department of Pathology, Oslo University Hospital, Oslo, Norway; 6 Department of Nuclear Medicine, Oslo University Hospital, Oslo, Norway; 7 Oslo and Akershus University College of Applied Sciences, Oslo, Norway; 8 Oslo Centre for Biostatistics and Epidemiology, Research Support Service, Oslo University Hospital, Oslo, Norway; 9 Department of Adult Epilepsy, National Centre for Epilepsy, Oslo University Hospital, Oslo, Norway

**Keywords:** Epilepsy surgery, Normal or non-specific histopathology, Normal MRI, Postsurgical seizure outcomes, Prognostic factors, Temporal lobe epilepsy

## Abstract

**Background:**

Seizure outcome following surgery in pharmacoresistant temporal lobe epilepsy patients with normal magnetic resonance imaging and normal or non-specific histopathology is not sufficiently presented in the literature.

**Methods:**

In a retrospective design, we reviewed data of 263 patients who had undergone temporal lobe epilepsy surgery and identified 26 (9.9%) who met the inclusion criteria. Seizure outcomes were determined at 2-year follow-up. Potential predictors of Engel class I (satisfactory outcome) were identified by logistic regression analyses.

**Results:**

Engel class I outcome was achieved in 61.5% of patients, 50% being completely seizure free (Engel class IA outcome). The strongest predictors of satisfactory outcome were typical ictal seizure semiology (*p* = 0.048) and localised ictal discharges on scalp EEG (*p* = 0.036).

**Conclusion:**

Surgery might be an effective treatment choice for the majority of these patients, although outcomes are less favourable than in patients with magnetic resonance imaging-defined lesional temporal lobe epilepsy. Typical ictal seizure semiology and localised ictal discharges on scalp EEG were predictors of Engel class I outcome.

## Introduction

The role of surgery as a treatment of pharmacoresistant temporal lobe epilepsy (TLE) is well established [12, 28, 39]. Between 70 and 90% of patients with hippocampus sclerosis (HS) on MRI concordant with other localisation methods may achieve seizure control following TLE surgery [17, 19, 30–32, 35, 40]. It is estimated that 20–30% of TLE patients have no lesion on MRI (N-MRI) [7, 35]. Unlike in patients with HS, the rate of seizure freedom following TLE surgery in this subgroup is lower and varies from 20 to 80% [2, 3, 5, 8, 21, 24, 34, 37].

There is no widely adopted standardised investigation protocol for TLE patients with N-MRI, and presurgical assessment remains challenging. Given the fact that medial temporal structures play an important role in memory function, physicians might be reluctant to recommend TLE surgery in settings of N-MRI. We present our experience with surgical treatment of this patient subgroup and a presurgical multimodal investigation process that has been used in the patient selection.

The aim of this study is to assess seizure outcomes following TLE surgery in patients with N-MRI in whom histopathological examination of the specimens was normal or non-specific (N-HP) and to identify potential prognostic factors for satisfactory seizure outcome (Engel class I).

## Methods

### Study setting

Between January 1999 and December 2013, 263 patients with pharmacoresistant TLE underwent unilateral temporal lobe surgery at our department. Twenty-six patients fulfilled the following inclusion criteria: (1) N-MRI, (2) N-HP and (3) seizure outcome assessed at 2-year follow-up. The data obtained were retrospectively reviewed and relevant variables were evaluated (Table 1).

**Table 1:** Univariate analysis of characteristics in 26 temporal lobe epilepsy patients with normal magnetic resonance imaging and without specific histopathology related to Engel class outcomes

Characteristic analysed	Patients	Engel class I	Engel class II-IV	*p* value
	*N* (%)	*N* (%)	*N* (%)	
Risk factors				
Febrile convulsions	2 (8)	1 (50)	1 (50)	0.211
Meningoencephalitis	2 (8)	1 (50)	1 (50)	
Head injury	5 (19)	5 (100)	0 (0)	
Unknown	17 (65)	9 (53)	8 (47)	
Age at seizure onset, median (min-max) (years)	13.5 (2–33)	15.5 (7–33)	9.5 (2–25)	0.031^a^
Duration of epilepsy, median (min-max) (years)	19.5 (4–47)	19.5 (4–47)	21 (5–46)	0.623
Age at surgery, median (min-max) (years)	37 (17–55)	38.5 (17–55)	31 (18–52)	0.182
Ictal seizure semiology				
Typical	19 (73)	14 (74)	5 (26)	0.036^a^
Atypical	7 (27)	2 (29)	5 (71)	
Seizure types				
Seizures without impairment of consciousness/awareness	1 (4)	1 (100)	0 (0)	0.329
Focal dyscognitive seizures	4 (15)	2 (50)	2 (50)	
Combination I^b^	9 (35)	7 (78)	2 (22)	
Combination II^c^	2 (8)	0 (0)	2 (100)	
Combination III^d^	10 (38)	6 (60)	4 (40)	
Seizure type frequency				
Without impairment of consciousness/awareness, median (min-max)/(months)	14 (4–300)	14 (4–300)	51 (8–140)	0.461
Focal dyscognitive seizures, median (min-max) (months)	8 (1–196)	5 (1–20)	8 (2–196)	0.080
Bilateral convulsive seizures, median (min-max) (years)	8 (1–52)	3.5 (1–36)	18 (4–52)	0.132
Interictal scalp EEG				
Localised discharges	8 (31)	6 (75)	2 (25)	0.347
Non-localised discharges	18 (69)	10 (56)	8 (44)	
Ictal scalp EEG				
Localised discharges	10 (38)	9 (90)	1 (10)	0.018^a^
Non-localised discharges	16 (62)	7 (44)	9 (56)	
Intracranial interictal EEG				
Localised discharges	12 (55)	8 (67)	4 (33)	0.211
Non-localised discharges	10 (45)	4 (40)	6 (60)	
Intracranial ictal EEG				
Medial discharges	12 (55)	6 (50)	6 (50)	0.783
Concurrent medial and lateral neocortical discharges	4 (18)	2 (50)	2 (50)	
Lateral neocortical discharges	6 (27)	4 (67)	2 (33)	
^18^F-FDG PET				
Localised	15 (88)	10 (67)	5 (33)	1.00
Non-localised (remote)	2 (12)	2 (100)	0 (0)	
^11^C-FMZ PET				
Localised	7 (78)	5 (71)	2 (29)	1.00
Non-localised (normal)	2 (22)	1 (50)	1 (50)	
Interictal SPECT				
Localised	13 (65)	5 (38)	8 (62)	0.350
Non-localised	7 (35)	5 (71)	2 (29)	
Ictal SPECT				
Localised	6 (75)	2 (33)	4 (67)	1.00
Non-localised	2 (25)	0 (0)	2 (100)	

^*11*^ *C-FMZ PET* [^11^C]flumazenil positron emission tomography, ^*18*^ *F-FDG PET* [^18^F]fluorodeoxyglucose positron emission tomography, *EEG* electroencephalography, *SPECT* single-photon emission computed tomography

^a^At the 0.05 level, higher median age at seizure onset, typical ictal seizure semiology and localised discharges on ictal scalp EEG are significantly associated with Engel class I outcome

^b^Combination I: combination of seizures without impairment of consciousness/awareness and focal dyscognitive seizures

^c^Combination II: combination of seizures without impairment of consciousness/awareness, focal dyscognitive seizures and bilateral convulsive seizures

^d^Combination III: combination of focal dyscognitive seizures and bilateral convulsive seizures

### Semiology and seizure classification

According to traditionally described TLE semiology [6, 27], we considered in this study that patients had typical ictal semiology of medial temporal seizure origin when presented with auras such as rising epigastric and gustatory sensations, oroalimentary automatisms, distortions, affective or experiential phenomena, followed by unilateral motor signs, such as distal limb automatism, ipsilateral contraction of the face or mouth, head deviation, behavioural arrest, alteration in consciousness, amnesia and lateral neocortical seizure origin when presented with staring, auditory and visual hallucinations, aphasia and focal sensory-motor phenomena. When patients presented with symptoms of early involvement of the frontal lobe, that is, non-verbal vocalisation, hypermotor seizures and asymmetric tonic posturing, then we considered semiology as atypical in this study.

The seizures were classified according to 2010 International League Against Epilepsy Commission proposal into: (1) seizures without impairment of consciousness/awareness, (2) focal dyscognitive seizures and (3) bilateral convulsive seizures [4].

### EEG protocol

**Fig. 1:**
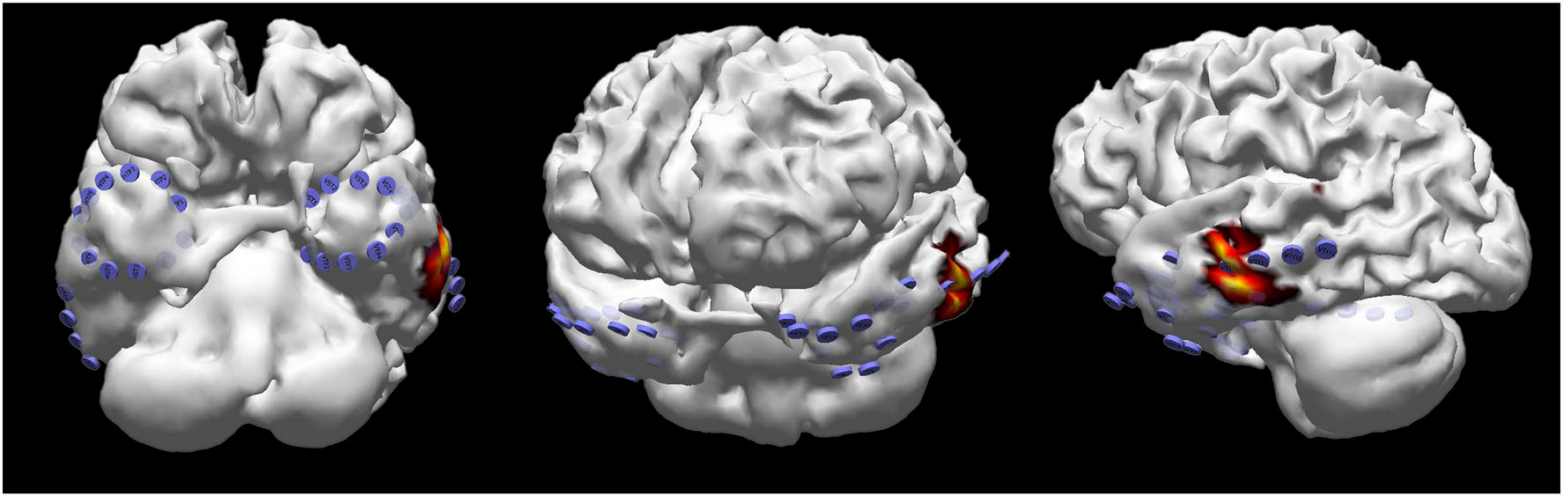
Location of the electrode contacts on superficial cortical anatomy of the temporal lobes, obtained by reconstruction of presurgical brain magnetic resonance imaging co-registered with computerised tomography scan after bilateral implantation of subdural strip electrodes, and localisation of the seizure onset zone on the laterobasal neocortex of the left temporal lobe, determined by the minimum norm solution of early ictal electroencephalographic activity, using methods as implemented in Curry software

Interictal and ictal scalp video EEGs were accomplished with Nervus/NicOne (Natus^©^, Middleton, WI, USA), with 25 or 64 scalp electrodes placed according to a 10–10 system. Patients with typical or atypical TLE semiology without clear localisation of the seizure onset zone on scalp EEGs and abnormality on functional neuroimaging (PET and/or SPECT) underwent intracranial EEG evaluation. However, patients with typical TLE semiology and clear localisation of the seizure onset zone on scalp EEGs concordant to findings on functional neuroimaging did not undergo intracranial EEG recording. According to this, 85% (22/26) of patients underwent intracranial EEG recordings. The placement of intracranial subdural electrodes (AD-TECH, Racine, WI, USA) was guided by ictal seizure semiology; the probable seizure onset area was determined by scalp EEGs and abnormality on functional neuroimaging. The location of subdural electrode contacts in relation to brain anatomy was determined with post-implantation CT scanning (GE, Little Chalfont, UK) co-registered to presurgical MRI using Curry software (Compumedics Neuroscan, Victoria, Australia). Also, MRI-based surface segmentations and border element method models were calculated to assess brain sources (Fig. 1).

Multiple ictal EEG recordings were obtained during seizures typical for the patient and the temporal lobe of seizure onset was defined, confirming TLE diagnosis. The seizure onset zone was defined by localisation of the electrographic seizure activity, early seizure spread (areas involved in seizure activity less than 10 s after the seizure onset) and dominant interictal epileptiform activity consisting of sharp and slow wave components. The results of scalp and intracranial EEG recordings were re-evaluated during multidisciplinary surgical planning conferences.

### MRI acquisition and assessment

Nineteen patients (73%) had 1.5-T MRI acquired using an Avanto (Siemens, Erlangen, Germany) and seven patients (27%) had 3-T MRI using an Achieva scanner (Phillips, Best, The Netherlands) with a 12-channel headcoil, performed according to the epilepsy protocol to obtain following sequences: 3D T1-weighted MP-RAGE (TR/TE 1900/3.46), flip angle of 15 degrees, slice thickness 1.25 mm and FOV 250; coronal FLAIR (TR/TI/TE 9000/2500/108) slice thickness 3 mm, FOV 230 and axial T2-weighted (TR/TE 4050/98) slice thickness 5 mm, FOV 230. All presurgical MRI studies were assessed qualitatively at the time of acquisition by neuroradiologists experienced in epilepsy imaging, reviewed again by epileptologists, neurosurgeons and epilepsy neuroradiologists on multidisciplinary patient management conferences, and described as completely normal.

### ^18^F-FDG PET, ^11^C-FMZ PET and SPECT evaluation

Sixty-five percent (17/26) of patients had interictal outpatient ^18^F-FDG PET (20-min static scan, 30 min after intravenous administration of 3 MBq/kg ^18^F-FDG), and 35% (9/26) had ^11^C-FMZ PET (40-min dynamic scan after intravenous administration of 4 MBq/kg ^11^C-FMZ) acquired using an ECAT Exact HR+ scanner (Siemens/CTI, Knoxville, TN, USA) or a Biograph-64 scanner (Siemens, Knoxville, TN, USA). Images were reconstructed using normalisation and attenuation-weighted ordered subset expectation maximisation (six iterations and eight subsets) after applying all appropriate corrections. The reconstruction process created a standard series of contiguous images oriented in the transaxial, coronal, sagittal and transtemporal planes. Images were evaluated visually and with a semiquantitative method with measurements of interhemispheric asymmetries. Routine EEG monitoring confirmed that no seizures occurred when acquisition was not performed.

Seventy-seven percent (20/26) of patients had interictal SPECT, and 40% of these (8/20) also had an ictal SPECT acquired by intravenous injection of 555–1000 MBq 99m Tc-HMPAO (hexamethylpropyleneamine oxime) or 99m Tc-ECD (ethyl cysteine dimer) using a Millennium VG Hawkeye dual-head γ-camera (GE, Waukesha, WI, USA) or an Infinia Hawkeye SPECT/CT (GE, Waukesha, WI, USA). To obtain ictal SPECT, the radiotracer was injected shortly after the seizure onset. Cerebral SPECT was performed 1 h later and images were reconstructed in the transaxial, coronal and sagittal planes. Three-dimensional projections were obtained and analysed visually and by statistical comparison to a normal database. Interictal SPECT study was performed in the same manner, except that the radiotracer was injected during the baseline conditions (no seizures within the last 24 h) and evaluated by direct comparison with the ictal SPECT study, when accessible.

The evaluation of ^18^F-FDG PET, ^11^C-FMZ PET and SPECT neuroimaging data were performed by experienced nuclear medicine physicians at the time of acquisition, without or with limited access to the other findings of the presurgical investigation, and all significant findings were reported. The findings of these neuroimaging studies were reviewed again on multidisciplinary patient management conferences.

For the purpose of this study, PET and SPECT abnormalities in the temporal lobe corresponding with seizure onset in the same region as defined by EEG were considered as localising. This was accepted regardless of remote ipsilateral and/or contralateral abnormalities. When findings of PET and SPECT neuroimaging were normal, without abnormalities in the temporal lobe of seizure onset, or showed only remote abnormalities outside the epileptogenic temporal lobe, they were considered as non-localising.

### Neuropsychological evaluation and Wada test

Memory test scores from presurgical neuropsychological assessments were obtained from 96% (25/26) of the patients. Three memory tests (two verbal and one visual) were performed with delayed recall scores (20–60 min): California Verbal Learning Test/California Verbal Learning Test, 2nd edition (CVLT) [9, 10], Luria Memory Words Test [1] and Figural Memory Test [18]. Verbal memory deficit was defined as a deficit in at least one of the verbal tests.

The intracarotid amobarbital/methohexital test (Wada test) was performed in 92% (24/26) of the patients. The language dominance was objectivised during hemi-anaesthesia by asking patients to name objects and perform simple commands. In the remaining 8% (2/26), the hemisphere dominance was determined by handedness. The surgery was performed in 13 patients on the language-dominant hemisphere (10 on the left and 3 on the right side) and in 13 patients on the language-non-dominant hemisphere (all on the right side). The memory was tested by showing ten objects under hemi-anaesthesia and then recounting in free recall when the patient had returned to normal condition and finally measured in a recognition test. All patients had a scalp EEG control during the procedure.

In both the presurgical neuropsychological assessment and Wada test, the memory deficit was defined as localising when concordant to the surgery side, i.e. verbal memory deficit and surgery on the language-dominant hemisphere or visual memory deficit and surgery on the language-non-dominant hemisphere. Presurgical memory test results were dichotomised into localising and non-localising and related to the seizure outcomes.

### Surgery, histopathology and outcomes

The surgical treatment decision and the extent of resection were based on the results of multimodal investigations. Twenty patients (77%) underwent standard anteromedial temporal lobectomy (AMTL). Four of them did not have intracranial EEG recordings, and surgical decision was based on congruent typical seizure semiology, localised PET or SPECT findings and localised ictal scalp EEG. The remaining 16 patients had intracranial ictal EEG discharges localised on either medial or concurrent medial and lateral neocortical structures (Table 1). The anterior temporal lobe was removed by en-block excision of the anterolateral neocortex, following the resection of the uncus and amygdala, and en-block resection of the parahippocampal gyrus and hippocampus. Both specimens were submitted to histopathological examination. Measured at the time of surgery, the extent of lateral neocortical resection along the long axis of the temporal lobe included the superior temporal gyrus limited to approximately 2 cm from the temporal tip and the middle, inferior and fusiform temporal gyri 3–5 cm on the dominant and 3.5–7 cm on the non-dominant side. Regardless of dominance, the hippocampus resection was approximately 2.5 to 3 cm from the pes hippocampi anterior tip in the temporal horn posteriorly along its length.

The remaining six patients (23%) had intracranial ictal EEG discharges localised on the lateral neocortex and underwent tailored anterolateral temporal lobe (ALTL) resection guided by intrasurgical electrocorticography to optimise the resection of electrographically active tissue. Measured at the time of surgery, the extent of tailored anterolateral neocortical resection along the long axis of the temporal lobe from the tip of temporal pole was 3.5–4 cm on the dominant and 5.5 cm on the non-dominant side. Thus, interictal discharges and early seizure-spread cortical areas were included in the resection.

Neuropathologists using a standard protocol for epilepsy surgery cases performed histopathological examinations of the resected specimens. In brief, surgical specimens from the lateral neocortex and hippocampus were fixed in formalin. The specimens were sectioned perpendicularly to the cortical surface into several tissue blocks. All the tissue blocks from the lateral neocortex and hippocampus were embedded in paraffin and stained with hematoxylin and eosin (H&E) and Luxol fast blue. Various immunostains (such as GFAP, myelin basic protein, phosphorylated neurofilament protein, NeuN, synaptophysin and alpha B-crystalline) were applied. The microscopic slides from surgical resections were re-reviewed by a neuropathologist in all cases. Out of 20 hippocampus specimens, 17 were completely normal and 3 had gliosis in the CA4 region. In 26 lateral neocortical specimens, 20 were completely normal and 6 had only reactive changes probably related to the intracranial EEG examinations (Table 2). These results were defined as N-HP.

**Table 2:** Univariate analysis of characteristics in 26 temporal lobe epilepsy patients with normal magnetic resonance imaging and without specific histopathology related to Engel class outcomes

Characteristic analysed	Patients	Engel class I	Engel class II–IV	*p* value
	*N* (%)	*N* (%)	*N* (%)	
Surgery types	26 (100)	16 (62)	10 (38)	
AMTL	20 (77)			
Language-dominant hemisphere	9 (45)	5 (56)	4 (44)	1.00
Language-non-dominant hemisphere	11 (55)	7 (64)	4 (36)	
ALTL	6 (23)			
Language-dominant hemisphere	4 (67)	3 (75)	1 (25)	1.00
Language non-dominant hemisphere	2 (33)	1 (50)	1 (50)	
Histopathological findings				
AMTL	20 (77)			
Normal	14 (70)	8 (57)	6 (43)	0.230
Reactive changes on lateral neocortex	3 (15)	3 (100)	0 (0)	
Gliosis in hippocampus	3 (15)	1 (33)	2 (67)	
ALTL	6 (23)			
Normal	3 (50)	2 (67)	1 (33)	1.00
Reactive changes	3 (50)	2 (67)	1 (33)	

*AMTL* anteromedial temporal lobectomy, *ALTL* anterolateral temporal lobe resection, *N* number of patients

The seizure outcomes at the 2-year follow-up were assessed according to the Engel classification [13] and presented in Table 3. The outcomes were dichotomised into satisfactory (Engel class I) and unsatisfactory (Engel class II–IV) subgroups.

**Table 3:** Seizure outcome score at 2-year follow-up in 26 temporal lobe epilepsy patients with normal magnetic resonance imaging and without specific histopathology

Engel class	AMTL = 20	ALTL = 6	Total = 26
	*N* (%)	*N* (%)	*N* (%)
I A	9 (45)	4 (66.6)	13 (50)
I B	3 (15)	0 (0)	3 (11.5)
II B	3 (15)	0 (0)	3 (11.5)
II D	1 (5)	0 (0)	1 (3.9)
III A	3 (15)	1 (16.7)	4 (15.4)
IVA	1 (5)	1 (16.7)	2 (7.7)

*AMTL* anteromedial temporal lobectomy, *ALTL* anterolateral temporal lobe resection, N-number of patients

### Statistical analysis

The data were described as number (percentage) or median (min-max) for categorical or continuous variables, respectively. Patients with satisfactory and unsatisfactory seizure outcomes were compared according to categorical variables with contingency tables using *χ* ^2^ or Fisher’s exact tests as appropriate and using the Mann–Whitney test for continuous variables.

To assess the possible influence of variables on the achievement of Engel class I outcome, univariate and multivariate logistic regression analyses were performed. The results of the analysis for each variable and its relation to Engel class I outcome in the final model were presented by logistic regression as an odds ratio (OR) with a 95% confidence interval (95% CI). Backward stepwise variable selection was performed using *p* ≤ 0.200 as the criterion. For all tests, *p* < 0.05 was considered to be a statistically significant difference between the groups. Stata/SE 13.0 (StataCorp LP, College Station, TX, USA) was used for all statistical analyses.

## Results

This cohort consisted of 26 patients (13 female) with a median age at surgery of 37 years (17–55). The seizure outcomes at 2-year follow-up are presented in Table 3. Satisfactory seizure outcome (i.e., free of disabling seizures) was achieved in 61.5% (16/26) of patients, specifically 60% (12/20) in AMTL and 66.6% (4/6) in ALTL subgroups. Unsatisfactory seizure outcome was achieved in 38.5% (10/26) of patients, specifically 40% (8/20) in the AMTL and 33.4% (2/6) in the ALTL subgroups. Moreover, AMTL performed on the language-dominant side resulted in satisfactory seizure outcome in 56% (5/9) and on the language-non-dominant side in 64% (7/11) of the patients. Furthermore, ALTL performed on the language-dominant side resulted in satisfactory seizure outcome in 75% (3/4) and on the language-non-dominant side in 50% (1/2) of the patients (Table 2).

Two-thirds of patients had seizure onset during childhood. The initial hospitalisation was preceded by bilateral convulsive seizures in 69% (18/26) of patients, but less obvious focal seizures might have been previously unrecognised. Six of 18 patients with history of bilateral convulsive seizures at the onset responded to antiepileptic drug therapy and did not experience this seizure type in the years until surgery. The most frequent seizure types at the time of surgery in our patient group were focal dyscognitive in 96% (25/26), followed by seizures without impairment of consciousness/awareness in 46% (12/26) and bilateral convulsive in 46% (12/26) of patients.

The neuropsychological assessment demonstrated generally good performance and low occurrence of memory deficit before surgery in our cohort. Mean CVLT delayed recall score was 12.3/16, mean Luria 10-word delayed recall score was 8.7/10, and mean figural memory delayed recall score was 9/10. Neuropsychological tests were localising in 40% (10/25) of the patients, more specifically in 56% (5/9) to the dominant and in 30% (3/10) to the non-dominant hemisphere for the AMTL subgroup, and in 25% (1/4) to the dominant and in 50% (1/2) to the non-dominant hemisphere for the ALTL subgroup. The Wada test localised memory deficit in 33% (8/24) of the patients, more specifically in 11% (1/9) to the dominant and in 60% (6/10) to the non-dominant hemisphere for the AMTL subgroup, and in 50% (1/2) to the non-dominant hemisphere for the ALTL subgroup. For both surgery types, there was no statistically significant difference in relation to Engel class outcomes when comparing localising to non-localising test results in both the dominant and non-dominant hemispheres.

Univariate analysis demonstrated that higher median age at seizure onset (*p* = 0.031), typical ictal seizure semiology (*p* = 0.036) and localised ictal discharges on scalp EEG (*p* = 0.018) were significantly associated with Engel class I outcome (Table 1).

Univariate logistic regression analysis demonstrated that there was an association between typical ictal seizure semiology and localised ictal discharges on scalp EEG with Engel class I outcome, OR 6.9; 95% CI 1.01–48.3; *p* = 0.048 and OR 11.6; 95% CI 1.17–114.3; *p* = 0.036, respectively. Higher age at seizure onset had a tendency to be associated with Engel class I outcome, OR 1.15; 95% CI 0.98–1.31; *p* = 0.055. The stepwise logistic regression multivariate model demonstrated that only localised ictal discharges on scalp EEG was an independent predictor of Engel class I outcome, OR 15.0; 95% CI 1.08–208.5; *p* = 0.044 (Table 4).

**Table 4:** Univariate and multivariate logistic regression analyses of characteristics related to Engel class I outcome

Characteristic analysed	Patient	Unit	Univariate analysis			Multivariate analysis		
	*N*		OR	(95% CI)	*p* value	OR	(95% CI)	*p* value
Age at seizure onset	26	Year	1.15	(0.98–1.31)	0.055			
Age at surgery	26	Year	1.05	(0.97–1.14)	0.250			
Ictal seizure semiology	26	If typical	6.90	(1.01–48.3)	0.048^a^	9.53	(0.84–108.1)	0.069
Focal dyscognitive seizures (frequency/month)	25	Number	0.90	(0.77–1.05)	0.180			
Bilateral convulsive seizures (frequency/year)	12	Number	0.95	(0.88–1.03)	0.222			
Ictal scalp EEG	26	If localised	11.6	(1.17–114.3)	0.036^a^	15.0	(1.08–208.5)	0.044^b^

*CI* confidence interval, *N* number, *OR* odds ratio

^a^At the 0.05 level, typical ictal seizure semiology and localised discharges on ictal scalp EEG are significantly associated with Engel class I outcome on univariate analysis

^b^At the 0.05 level, localised discharges on ictal scalp EEG are significantly associated with Engel class I outcome on multivariate analysis

## Discussion

### Outcomes

The choice of optimal treatment strategy for pharmacoresistant TLE patients with N-MRI remains challenging. In general, satisfactory seizure outcome is less likely achieved than in patients with lesional TLE. However, the rate of seizure freedom following TLE surgery in patients with N-MRI differs among studies. A possible explanation might be that patients with TLE and N-MRI constitute a very heterogeneous group. In fact, some of them may be truly non-lesional (N-HP), while others may have different histopathological findings as demonstrated in several studies [14, 22, 29, 33, 34]. Due to different ratios of histopathological substrates in each study, the seizure outcomes between various reports are difficult to compare.

In a meta-analysis from 2010 by Téllez-Zenteno et al. [35], the proportion of seizure freedom following surgery in non-lesional TLE was 51% of 226 patients when MRI was used and 36% of 172 patients when histopathology was used to define non-lesional status. In another meta-analysis from 2016, Wang et al. [38] reported a seizure freedom rate of 54.9% in the pooled analysis of 82 patients with N-MRI and N-HP who underwent temporal lobe surgery. However, the later meta-analysis included a study [37] that was lacking histopathological results of lateral neocortical specimens.

To assess seizure outcomes following TLE surgery in a truly non-lesional subgroup, we included 26 adults with normal or non-specific histopathology. In the current study, complete seizure freedom (Engel class IA) was achieved in the AMTL subgroup in 45% of patients, whereas in the ALTL subgroup in 66.6% of patients (Table 3). An explanation of favourable seizure outcome in ALTL subgroup of patients in our series might be related to individualised tailored resection increasing the probability of a complete resection.

The result from the AMTL subgroup in this study is similar to the findings of Benedetti-Isaac et al. [3] who reported the seizure freedom rate, at mean 6.5-year follow-up, in 42.9% of patients with N-MRI and N-HP who underwent the same surgical procedure. Thus, these promising results are particularly important in the current era when epilepsy surgery is underutilised, especially in TLE subgroup with N-MRI.

### Predictors of satisfactory outcome

Typical ictal seizure semiology in the majority of TLE patients with N-MRI and N-HP in this series mainly had the same features traditionally described with medial rather than lateral neocortical seizure onset. An explanation for this might be that ictal clinical seizure semiology reflects common seizure propagation involving the medial temporal lobe pathways. We found on univariate analysis that the typical ictal TLE semiology was a potential prognostic indicator of satisfactory outcome (Table 1), and its significance was confirmed on univariate logistic regression analysis (Table 4). This finding suggests that clinical seizure characteristics are a critical part of presurgical evaluation in this patient population, in accordance with a recently published report [22].

In our cohort, patients with unsatisfactory outcome subgroups had higher presurgical seizure burdens than those in the satisfactory outcome subgroup, but the difference was not significant on univariate analysis (Table 1). Furthermore, we found that the burdens of focal dyscognitive and bilateral convulsive seizures on logistic regression analysis were not prognostic indicators of satisfactory outcome (Table 4). These findings were not surprising because the high presurgical seizure burden and history of bilateral convulsive seizures in TLE patients with N-MRI were considered to be indicators of a more active and extensive epileptogenic network, possibly beyond the temporal lobe and/or involving the contralateral side, and are therefore considered predictors of poor seizure outcomes [14].

Wang et al. [38] found that a shorter epilepsy duration predicted better seizure outcome following surgery; however, our findings could not corroborate this. On the other hand, we found by univariate analysis that patients with older age at seizure onset had a higher probability of achieving Engel class I outcome (Table 1), but the significance was not confirmed on logistic regression analysis (Table 4), in accordance with one previously published report [37].

Not surprisingly, we found on univariate analysis that ictal localised discharges on scalp EEG were identified as potential prognostic indicators of satisfactory outcome (Table 1). It was a reliable predictor of seizure freedom following TLE surgery in this patient population on logistic regression analysis (Table 4), which is in agreement with earlier observations [11, 17, 21, 34, 38].

Some authors argued for the use of intracranial EEG because this technique optimises the planning of epileptic zone resection extent, thereby reducing the residual epileptogenic tissue, which may contribute to postsurgical seizures [22, 36]. Other authors recognised only a limited additive value of intracranial EEG [23, 37]. In our cohort, intracranial EEG localised the seizure onset precisely and contributed to the decision to tailor resections in some cases, but it failed to predict satisfactory outcomes on univariate analysis (Table 1).

Interictal ^18^F-FDG PET is a widely used neuroimaging tool that might be useful for lateralisation and localisation of the seizure onset in TLE patients with N-MRI, making an a priori hypothesis about subsequent intracranial electrode placement possible [14, 15, 20, 37]. As ^18^F-FDG PET measures glucose metabolism related to the synaptic and neuronal activity of the brain tissue [26], it was not surprising that the majority of patients in our cohort (88%) exhibited localised abnormality of the temporal lobe. However, our study demonstrated that this functional imaging failed to predict satisfactory outcome (Table 1), which is in agreement with previously published data [2, 14, 37].

^11^C-FMZ PET is an index of brain benzodiazepine receptor binding potential and may represent neuronal loss or receptor changes related to epileptogenicity. It demonstrated localised abnormality on the epileptogenic temporal lobe in 78% of patients. However, ^11^C-FMZ PET findings were not prognostic indicators of satisfactory outcome in our cohort (Table 1). According to our best knowledge, there are no previous reports on ^11^C-FMZ PET results in this patient population.

Interictal and ictal SPECTs demonstrate perfusion changes in the brain tissue independent of structural abnormalities and are utilised for localisation of the seizure onset in TLE patients with N-MRI [25, 37]. The majority of our patients had interictal SPECT and only a small proportion had ictal SPECT. These functional imaging techniques failed to predict satisfactory outcome in this patient population (Table 1), which is in agreement with previously published data [15, 16, 34, 37]. On the contrary, it was demonstrated that ictal-interictal subtraction SPECT co-registered to MRI (SISCOM) excellently localised the ictal seizure onset zone in TLE patients with N-MRI and provided higher predictive values of good seizure outcome [2].

### Limitations

The sample size and retrospective nature of the study must be considered limitations. Furthermore, although the same imaging and EEG protocols were used in the majority of patients, there were different scanners and EEG devices in use over the 15-year-long time period of data collection. Another potential criticism may be addressed to the image quantification and post-processing techniques that were not used, adding uncertainty to proper localisation of the seizure onset for those who did not achieve seizure freedom. This study had sufficient power to detect two predictors of satisfactory postsurgical outcome; however, the width of the confidence intervals may indicate the lack of precision in the analysis.

## Conclusion

We report the postsurgical seizure outcome and predictors of satisfactory seizure outcome in a group of pharmacoresistant TLE patients with N-MRI and N-HP. Our results suggest that satisfactory seizure outcome may be achieved in the majority of patients with N-MRI presenting with typical ictal seizure semiology and localised ictal discharges on scalp EEG. These promising results encourage early referral of pharmacoresistant TLE patients with N-MRI to a comprehensive epilepsy centre for detailed surgical evaluation, which might include intracranial EEG recording. Further research to validate our results using image quantification and post-processing techniques with respect to their ability to demonstrate and define seizure onset zones and their impact on postsurgical outcomes are needed.

## Abbreviations

^11^C-FMZ PET: [^11^C]flumazenil positron emission tomography
^18^F-FDG PET: [^18^F]fluorodeoxyglucose positron emission tomography
ALTL: Anterolateral temporal lobe resection
AMTL: Anteromedial temporal lobectomy
CT: Computed tomography
CVLT: California Verbal Learning Test/California Verbal Learning Test, 2nd edition
EEG: Electroencephalography
HS: Hippocampal sclerosis
MRI: Magnetic resonance imaging
N-HP: Normal or non-specific histopathology
N-MRI: Normal magnetic resonance imaging
SISCOM: Ictal-interictal subtraction SPECT co-registered to MRI
SPECT: Single-photon emission computed tomography
TLE: Temporal lobe epilepsy
